# Comparison of Extenders With the Addition of Egg Yolk for Cooling Alpaca Sperm Obtained From Deferent Ducts

**DOI:** 10.3389/fvets.2020.597954

**Published:** 2020-11-30

**Authors:** Mariana Lucía Bertuzzi, Edita Yola Torres, Teodosio Huanca, Deborah Neild, María Ignacia Carretero

**Affiliations:** ^1^Facultad de Ciencias Veterinarias, INITRA, Cátedra de Teriogenología, Universidad de Buenos Aires, Ciudad Autónoma de Buenos Aires, Buenos Aires, Argentina; ^2^Consejo Nacional de Investigaciones Científicas y Técnicas (CONICET), Ciudad Autónoma de Buenos Aires, Buenos Aires, Argentina; ^3^Laboratorio de Reproducción Animal, Facultad de Medicina Veterinaria y Zootecnia, Universidad Nacional del Altiplano Puno, Puno, Peru; ^4^Instituto Nacional de Innovación Agraria (INIA), Centro de Investigación y Producción Quinsachata, Puno, Peru

**Keywords:** extenders, cooling, egg yolk, sperm, alpaca, deferent ducts

## Abstract

The use of non-commercial and commercial extenders for cooling alpaca sperm has already been reported, the latter showing certain advantages over the first. The Andromed® (AM) extender was created for use in ruminants and has also been tested in ejaculated and epididymal alpaca sperm. According to the manufacturer, this extender does not need the addition of egg yolk (EY); however, it is known that the addition of EY to some extenders improves the preservation of cooled sperm. The objective of this study therefore was to compare a non-commercial extender (Tris) with the addition of EY vs. the commercial extender AM with and without the addition of EY, for cooling alpaca sperm obtained from diverted deferent ducts. Fifteen pools of deferent duct sperm were formed using samples from two or three different males for each. Each sperm pool was evaluated and then divided into three aliquots that were diluted to a final concentration of 30 × 10^6^ sperm ml-1 (0 h) with either: (1) Tris with 20% EY (T-EY), (2) AM, or (3) AM with 20% EY (AM-EY). Samples were cooled to 5°C and the following sperm parameters were evaluated after 24 and 48 h of storage: motility, viability, membrane function, acrosome integrity, morphology, and chromatin condensation. Motility was also evaluated after 72 h of storage. The samples that best preserved progressive and total sperm motility at the 24 and 48 h evaluation periods were the ones diluted with AM-EY, observing that with this extender these motility patterns decreased significantly after 72 h of storage compared to time 0 h (*p* < 0.05). A significant decrease (*p* < 0.05) in total and progressive motility was observed at 48 h for the T-EY and AM extender compared to 0 h. AM was the only extender in which the percentages of viable sperm decreased significantly (*p* < 0.05) after 48 h of conservation. For the rest of sperm parameters evaluated, no significant differences were observed between any of the extenders at any evaluation time. The Andromed® extender with the addition of 20% EY could be an alternative option for cooling alpaca sperm obtained from deferent ducts.

## Introduction

One of the most important reproductive biotechnologies for production is artificial insemination (AI) which, when compared to natural mating, maximizes the use of genetically superior males and achieves a rapid genetic progress. However, in South American Camelids (SACs) this biotechnology is currently limited to use with diluted raw semen, with a maximum pregnancy rate of 77% in experimental centers and no higher than 50% in private establishments (1, 2). This methodology using raw semen presents some disadvantages such as inadequate laboratory environments for conditioning the samples and the need to transport the males to the site of insemination due to the very large distances between farms. As an alternative to this, cooling semen for subsequent AI presents many advantages, such as prevention and control of venereal diseases and not requiring the presence of the breeding males on the premises, thus eliminating the costs of upkeep and providing greater security for the animals because transport is avoided. However, pregnancy rates obtained when using cooled SAC semen preserved for 24 h have been lower than those using diluted raw semen [0–33%: (2–4)]. Murillo et al. (5) obtained slightly higher pregnancy rates (40–46.7%) when using alpaca semen obtained by post-mating aspiration from the female's vagina and cooled with Andromed® and Triladyl®. However, they only preserved the samples for 6 h.

It has been postulated that the lower pregnancy rates obtained when inseminating with SAC cryopreserved semen could be linked to the rheological characteristics, such as thread formation and the high structural viscosity, of semen from these species (6–9) because these characteristics prevent an efficient homogenization of the ejaculate with the diluents (9–11). Hence, collecting sperm from the deviated deferent ducts is an interesting technique to apply in SACs as the samples thus obtained do not show thread formation, permitting a better homogenization with the extenders and as a result could have a beneficial effect on the cryopreservation of the samples.

The use of commercial extenders to cryopreserve sperm samples presents certain advantages over non-commercial ones: they require less equipment and supplies, are easy to prepare and are less variable in composition, which allows more repeatable results to be obtained. In alpacas, cooling sperm has been carried out using both commercial and non-commercial extenders (3–5, 12–14). The non-commercial extender most used for cooling alpaca sperm is Tris with the addition of different types of egg yolk (4) or membrane protective agents (13). Within commercial extenders, Triladyl® (3, 5), Biladyl® and Androhep® (12) have been assayed for cooling alpaca semen. Furthermore, the Andromed® (AM) extender, although created to be used in ruminants, has also been tested in alpaca sperm, both from ejaculates and from epididymis (5, 14). According to the manufacturers, one of the advantages of the AM extender is that it does not require the addition of EY thus avoiding the use of a product of animal origin. However, the addition of egg yolk to extenders is known to exert a protective effect on sperm, especially those of SACs, and this effect is attributed to its ability to interact with the plasma membrane lipid bilayer (15, 16) preventing the phase transition events of membrane lipids (16, 17). To our knowledge, no commercial extenders have been used to maintain cooled sperm obtained from deviated deferent ducts, neither have the effects of the addition of EY to commercial extenders been evaluated in these species.

In this context, the objective of this study was to compare a non-commercial extender (Tris) with the addition of EY vs. the commercial extender AM with and without the addition of EY, in cooled alpaca sperm obtained from diverted deferent ducts.

## Materials and Methods

### Reagents

Hoescht 33342, Propidium iodide, and the reagents for the PBS medium and for the HOS test were purchased from Sigma Chemicals (Sigma Aldrich, Buenos Aires, Argentina). Coomassie blue was purchased from Bio-Rad, California, USA.

### Experimental Design

Fifteen pools of deferent duct sperm were formed using samples from two or three different males each. After collection, sperm motility, concentration, membrane integrity (viability), membrane function and acrosome integrity, morphology and chromatin condensation were evaluated in each pool and the samples were then divided into 3 aliquots. With the objective of standardizing sperm concentration, each aliquot was diluted to 30 × 10^6^ sperm/ml with the following extenders, previously warmed to 37°C: (1) Tris with 20% EY (T-EY), (2) Andromed® (AM), and (3) AM with 20% EY (AM-EY). Once diluted, motility and viability were again evaluated, and this was considered time 0 h. After this the samples were cooled to 5°C in a controlled cooling device with a digital thermometer, that brought temperature down from 37 to 5°C, over a lapse of 7 h, and then maintained temperature at 5°C for a period of 72 h. At 24 and 48 h of conservation, an aliquot of each sample was warmed to 37°C for 15 min and the sperm parameters of motility, viability, membrane function and acrosome integrity, morphology and chromatin condensation were evaluated. Finally, at 72 h, motility was again evaluated.

### Animals and Location

The study was carried out during the month of October, in the Quimsachata experimental station, owned by the National Institute of Agricultural Innovation (Instituto Nacional de Innovación Agraria; INIA), which is located in the Santa Lucia district in the Lampa province of the Puno region, Peru. This is an agroecological area of the Dry Puna and is situated 4200 meters above sea level (15°41′39″ latitude and 70°36′24″ longitude). Five adult Huacaya-breed alpaca (*Vicugna pacos*) males, between 4 and 10 years of age, were used. Their deferent ducts were surgically deviated using the technique described by Pérez and Apaza (18). Briefly, the males were sedated with acepromazine and were held in dorsal recumbency. The surgical field in the inguinal region was prepared, local anesthesia administered and an incision 4 cm from the base of the penis was made to locate the deferent duct. After dissecting 7 cm of deferent duct, the freed extremity was redirected below the subcutaneous, fixed to the inner face of the femoral region and protected by a temporary patch. The animals were kept in pens, consisting of covered grazing areas with natural grasses, and had free access to water throughout the study.

### Obtaining Sperm From the Deferent Ducts

Sperm samples were obtained following a modification of the method described by Pérez et al. (19). Briefly, the male was held in lateral recumbency and the area surrounding the fistula was disinfected. A soft massage using fingertips was applied with a caudo-craneal direction, from the tail of the epididymis, helping move the sperm toward the exit of the fistula and once a white droplet (spermatic portion) was observed, it was gently aspirated with a 10 μl micropipette using tips that had already been soaked in Tris extender. The sperm sample was then deposited in a tube with Tris previously warmed to 37°C and kept in a water bath at the same temperature. Once the sperm extraction was completed, the surrounding area was once again gently disinfected, dried and covered with solid Vaseline. The collection maneuver was repeated in two or three other males and a pool was made with the different samples. Each deferent duct from every male was collected every 2 days to avoid sperm accumulation and resulting cell death.

### Evaluation of Sperm Parameters

The following sperm parameters were evaluated: motility, concentration, membrane integrity (viability), membrane function and acrosome integrity, morphology and chromatin condensation.

Sperm motility was evaluated using a phase contrast microscope (100×) and a warm stage (37°C). The patterns observed were: oscillatory motility (OM), progressive motility (PM) and circular motility (CM). In addition, total sperm motility was determined (TM = OM + PM + CM). Spermatozoa with OM are those that move in their place, without advancing whereas sperm with PM move in a straight line or describing large circles. Lastly, sperm with CM are those that move in very tight circles.

Sperm concentration was calculated using a Neubauer hemocytometer.

To evaluate membrane integrity, 12.5 μl of the sample were incubated at 37°C for 10 min in 127 μl of the staining medium, which contained 2 μl of a Hoescht 33342 solution (10 μg/ml in PBS) and 125 μl of a saline medium [described by Harrison and Vickers (20)]. After the first 10 min of incubation, 2 μl of a solution of Propidium iodide (0.5 mg/ml in isotonic saline solution) was added and the sample was incubated a further 10 min at 37°C. A minimum of 200 sperm were evaluated per sample, using an epifluorescence microscope with UV filter (400X) (Zeiss AxioScope). Sperm that fluoresced blue were classified as viable with intact membranes, and those that had a pink fluorescent nucleus were classified as non-viable, with damaged membranes.

To evaluate membrane function and acrosome integrity, the dual technique of combining the HOS test with Coomassie blue (CB) stain was carried out according to Carretero et al. (21). Briefly, first the HOS test was carried out on the sperm samples and after the 20 min of incubation, samples were fixed with 4% paraformaldehyde in PBS, incubated and centrifuged at 800 g and room temperature for 10 min. The pellet was re-suspended in 100 μl of PBS, microdroplets of the suspension were placed on slides and left to dry after which they were stained with 0.22% CB and observed using light microscopy (1000x). A total of 200 sperm were evaluated and classified into one of the following categories: (1) sperm with a functional membrane (HOS+: with tail swelling) and with an acrosome (CB+: violet acrosome staining), (2) sperm with a functional membrane (HOS+: with tail swelling) and without an acrosome (CB−: no acrosome staining), (3) sperm with a non-functional membrane (HOS−: no swelling) and with an acrosome (CB+: violet acrosome staining), and (4) sperm with a non-functional membrane (HOS−: no swelling) and no acrosome (CB−: no acrosome staining).

Sperm morphology was evaluated using phase contrast microscopy (1000x), after placing a 10 μl drop of sample on a glass slide with a coverslip. For each sample, 200 spermatozoa were evaluated, and were classified into one of the following categories: normal, abnormal head, detached head, abnormal midpiece, abnormal tail and cytoplasmic droplet. Thus, percentages of spermatozoa with normal or altered morphology were determined.

The degree of chromatin condensation was evaluated with the Toluidine Blue (TB) stain according to Carretero et al. (22). Briefly, samples were smeared onto clean slides, fixed with ethanol 96° for 2 min and stained during 5 min with 0.02% TB. Preparations were observed directly under immersion oil using a phase contrast microscope (1000x), evaluating a minimum of 200 spermatozoa per smear. Sperm were classified into three patterns: light blue (TB negative), light violet (TB intermediate) and dark blue-violet (TB positive). TB negative sperm were considered to have normal, highly condensed chromatin and TB intermediate + TB positive sperm were considered to have decondensed chromatin (between moderate and high decondensation).

### Statistical Analysis

Statistical analysis was carried out using InfoStat software (Student Version) (https://www.infostat.com.ar/index.php?mod=page&id=15). In all cases normal distribution and homogeneity of variances of the data were corroborated using Shapiro-Wilk's Normality test and an ANOVA, respectively. The level of significance was set at 0.05 for all analysis.

Because sperm motility and chromatin condensation did not show a normal distribution or homogeneity of variances, these data were compared using a Kruskal–Wallis test.

To compare viability of collected sperm before dilution to the samples diluted at time 0 h, an ANOVA was applied using a factorial design with one factor with 4 levels (collected sperm before dilution, T-EY 0 h, AM 0 h and AM-EY 0 h).

To compare viability between diluted cooled samples, an ANOVA was applied using a factorial design with two factors: extender (with 3 levels: T-EY, AM and AM-EY) and time (with 3 levels: 0, 24 and 48 h).

For analyzing the parameters of sperm morphology and of the HOS/CB test (sperm membrane function and acrosome), an ANOVA was applied using a factorial design with one factor with 7 levels (collected sperm prior to dilution, T-EY 24 h, AM 24 h, AM-EY 24 h, T-EY 48 h, AM 48 h and AM-EY 48 h).

## Results

Oscillatory, progressive, circular and total motility in the samples diluted at 0 h were not different to that of sperm collected from the deviated deferent ducts prior to dilution (*p* > 0.05). The samples that best preserved progressive and total sperm motility at 24 and 48 h evaluation times were the ones diluted with AM-EY (*p* > 0.05), observing that with this extender these motility patterns decreased significantly only after 72 h of storage compared to time 0 h (*p* < 0.05). Whereas a significant decrease (*p* < 0.05) in total and progressive motility was observed at 48 h for the T-EY and AM extender compared to time 0 h. It is interesting to highlight that in the samples cooled in AM, mainly oscillatory motility was observed at 48 and 72 h of conservation whereas, in the samples cooled in T-EY and AM-EY the motility pattern observed was mostly progressive, similar to the collected sample (54.5 ± 16.1%) (Table 1).

**Table 1:** Oscillatory motility (OM), progressive motility (PM), circular motility (CM), and total motility (TM) in alpaca spermatozoa collected from deviated deferent ducts and diluted (0 h) in Tris with 20% egg yolk (Tris-EY), Andromed® (AM) and AM with 20% egg yolk (AM-EY) evaluated at 24, 48 and 72 h of storage at 5°C.

	**OM (%)**	**PM (%)**	**CM (%)**	**TM (%)**
Tris-EY 0 h	1.1 ± 2.7^a^	52.0 ± 11.3^a^	0.2 ± 0.7^a^	53.3 ± 10.2^a^
AM 0 h	1.8 ± 3.0^a^	47.5 ± 13.5^a^	1.4 ± 2.1^ab^	50.7 ± 10.4^a^
AM-EY 0 h	0.3 ± 0.8^a^	53.4 ± 9.9^a^	0.3 ± 0.7^ab^	54.0 ± 9.1^a^
Tris-EY 24 h	2.1 ± 2.8^a^	41.8 ± 8.8^a^	0.8 ± 1.5^ab^	44.7 ± 6.6^ab^
AM 24 h	17.7 ± 12.8^b^	19.8 ± 17.2^b^	2.1 ± 3.1^ab^	39.6 ± 8.5^ab^
AM-EY 24 h	1.0 ± 1.8^a^	46.2 ± 8.7^a^	0.8 ± 1.4^ab^	48.0 ± 7.7^a^
Tris-EY 48 h	3.7 ± 4.1^a^	29.4 ± 12.5^b^	4.4 ± 3.1^bc^	37.5 ± 10.8^bc^
AM 48 h	17.4 ± 10.8^b^	9.1 ± 11.6^b^	2.1 ± 3.2^ab^	28.8 ± 8.5^bc^
AM-EY 48 h	2.0 ± 3.7^a^	40.9 ± 11.6^ab^	3.9 ± 3.0^ab^	46.8 ± 9.1^ab^
Tris-EY 72 h	2.7 ± 4.1^a^	21.7 ± 10.1^b^	8.6 ± 6.8^c^	33.0 ± 9.7^c^
AM 72 h	20.0 ± 11.3^b^	0.7 ± 1.6^c^	1.0 ± 2.1^ab^	21.7 ± 11.8^c^
AM-EY 72 h	2.1 ± 2.3^a^	35.7 ± 8.5^b^	3.2 ± 3.3^ab^	41.0 ± 7.7^b^

*Values are expressed as mean ± SD (n = 15)*.

a, b, c *Different letters between rows within each column, indicate significant differences (p < 0.05)*.

**Figure 1:**
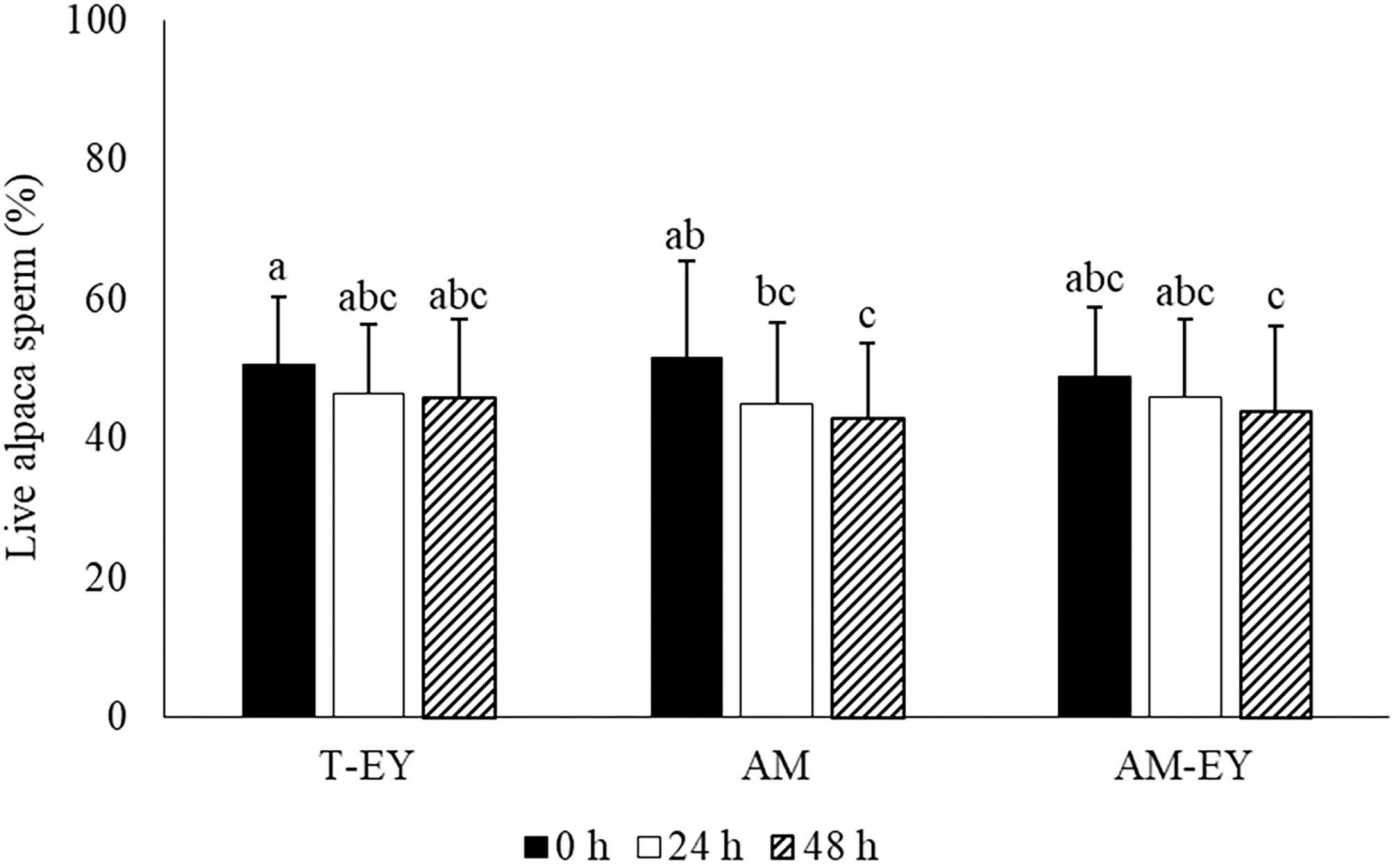
Percentages of live sperm in alpaca samples collected from deviated deferent ducts and diluted (0 h) in Tris with 20% egg yolk (T-EY), Andromed® (AM) and AM with 20% egg yolk (AM-EY) evaluated at 24 and 48 h of storage at 5°C (*n* = 15). ^a, b, c^Different letters indicate significant differences between samples and evaluation times (*p* < 0.05).

Percentages of viable sperm in the samples diluted at 0 h were not different to that of sperm collected from the deviated deferent ducts prior to dilution (*p* > 0.05). AM was the only extender in which the percentages of viable sperm decreased significantly (*p* < 0.05) after 48 h of conservation compared to 0 h (Figure 1). However, no significant differences were observed in viability between the extenders at any of the different evaluation times (Figure 1).

**Figure 2:**
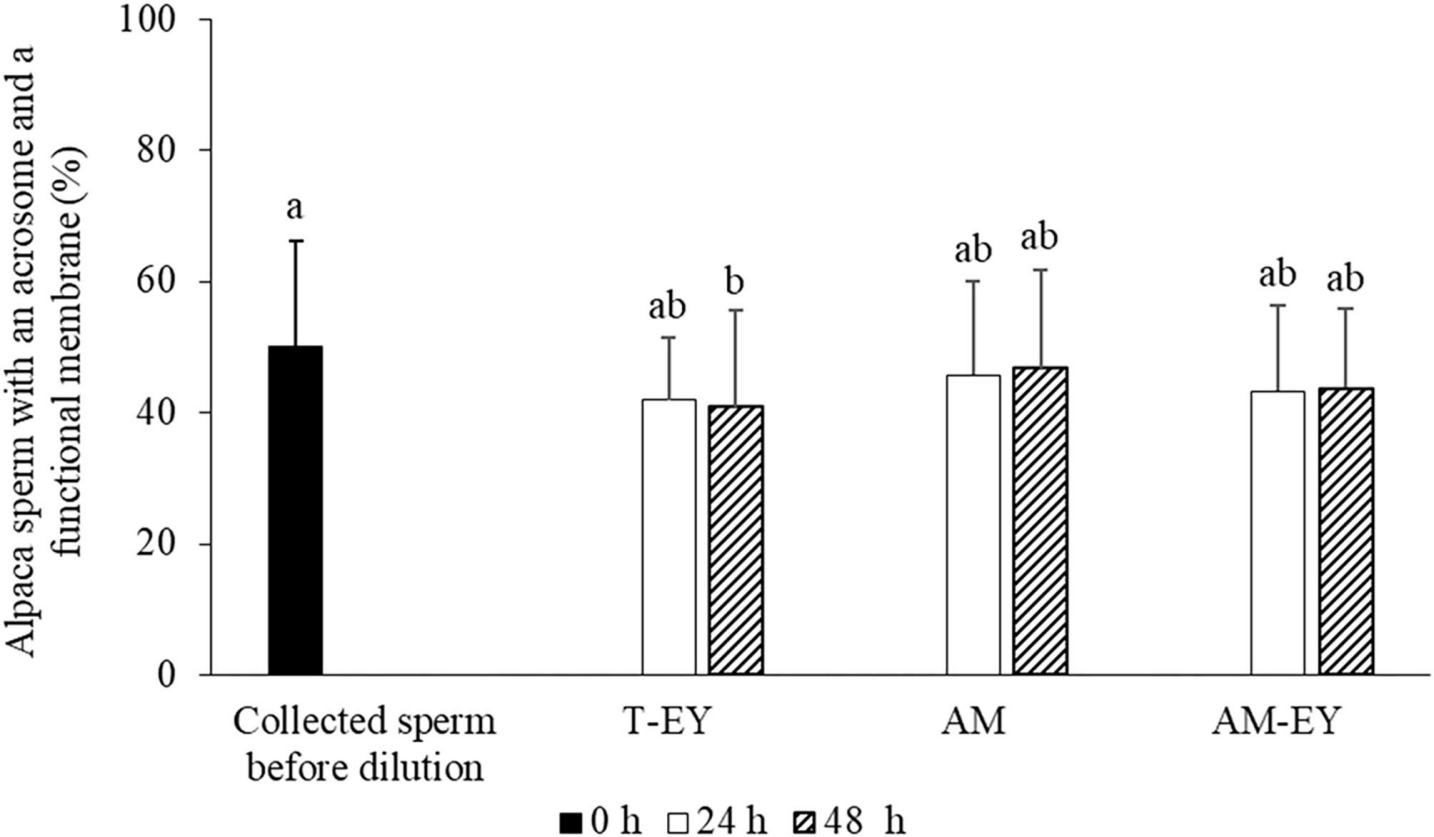
Percentages of sperm with an acrosome and a functional membrane in alpaca samples collected from deviated deferent ducts and cooled to 5°C in Tris with 20% egg yolk (T-EY), Andromed® (AM) and AM with 20% egg yolk (AM-EY), evaluated at 24 and 48 h of storage (*n* = 15). ^a, b^Different letters indicate significant differences between samples and evaluation times (*p* < 0.05).

In addition, no significant differences (*p* > 0.05) were observed in the percentages of sperm with functional membranes and presence of acrosomes (HOS+/CB+) between collected sperm before dilution and cooled samples at 24 and 48 h, with the exception of cooled samples diluted with T-EY which presented a significant decrease of HOS+/CB+ sperm at 48 h compared to collected sperm before dilution (Figure 2).

**Figure 3:**
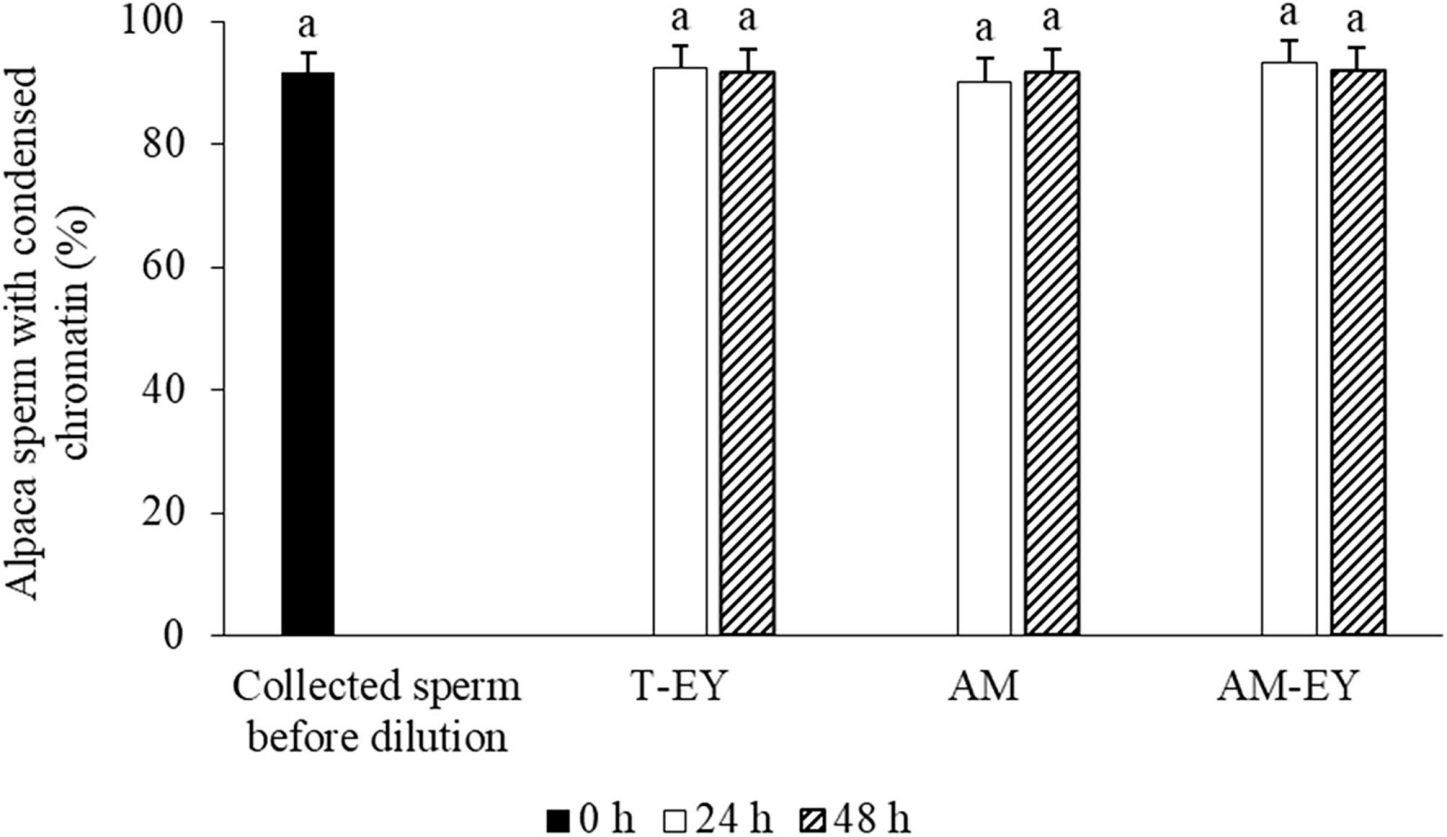
Percentages of sperm with condensed chromatin in alpaca samples collected from deviated deferent ducts and cooled to 5°C in Tris with 20% egg yolk (T-EY), Andromed® (AM) and AM with 20% egg yolk (AM-EY) evaluated after 24 and 48 h of storage *(n* = 15). ^a^Indicates absence of significant differences (*p* > 0.05).

No significant differences were observed over time in the percentage of sperm with normal morphology (Table 2) and condensed chromatin (Figure 3) nor between any of the extenders at any of the evaluation times (*p* > 0.05).

**Table 2:** Sperm morphology in samples collected from alpaca deviated deferent ducts and in samples cooled to 5°C in Tris with 20% egg yolk (T-EY), Andromed® (AM) and AM with 20% egg yolk (AM-EY) evaluated after 24 and 48 h of storage.

	**Normal**	**Abnormal**	**Detached**	**Abnormal**	**Abnormal**	**Citoplasmic**
	**sperm (%)**	**heads (%)**	**heads (%)**	**tails (%)**	**midpieces (%)**	**droplets (%)**
Collected sperm before dilution	29.3 ± 9.5^a^	14.4 ± 4.1^a^	11.1 ± 5.7^a^	16.7 ± 7.4^a^	14.4 ± 3.2^a^	14.1 ± 3.7^a^
Tris-EY 24 h	30.4 ± 11.6^a^	16.0 ± 3.0^a^	9.5 ± 7.0^a^	17.8 ± 8.9^a^	14.3 ± 3.4^a^	12.0 ± 3.7^ab^
AM 24 h	29.7 ± 10.3^a^	16.5 ± 5.2^a^	10.7 ± 8.1^a^	16.2 ± 5.3^a^	13.2 ± 2.0^a^	13.7 ± 4.3^a^
AM-EY 24 h	29.4 ± 9.0^a^	17.5 ± 4.5^a^	11.4 ± 8.8^a^	16.6 ± 7.7^a^	13.8 ± 3.5^a^	11.3 ± 4.2^ab^
Tris-EY 48 h	29.5 ± 9.0^a^	17.9 ± 5.3^a^	12.8 ± 11.3^a^	17.2 ± 5.5^a^	12.8 ± 3.8^a^	9.8 ± 3.7^b^
AM 48 h	29.1 ± 7.4^a^	15.6 ± 5.5^a^	11.6 ± 11.2^a^	16.8 ± 6.3^a^	12.9 ± 4.7^a^	14.0 ± 7.4^a^
AM-EY 48 h	29.7 ± 8.4^a^	16.8 ± 6.2^a^	11.9 ± 11.1^a^	15.4 ± 6.3^a^	15.2 ± 4.8^a^	11.0 ± 4.1^ab^

*Values are expressed as mean ± SD (n =15)*.

a, b *Different letters between rows indicate significant differences for each variable analyzed (p < 0.05)*.

## Discussion

Given the current importance of the use of commercial extenders for cryopreserving the male gamete, the objective of this study was to compare a non-commercial extender to a commercial one for cooling alpaca sperm obtained from deviated deferent ducts. In this context, we observed that the AM extender with the addition of EY was the one that presented the highest percentage of sperm with total and progressive motility after 48 h and even after 72 h of conservation. The total motility observed (47% after 48 h) was higher than that reported by other authors that assayed commercial extenders for cooling alpaca sperm. For example, Morton et al. (12) obtained 36% and 16% TM at 24 and 48 h, respectively, in alpaca samples cooled in Biladyl, an extender that has EY in its preparation. Others, such as Vaughan et al. (3) and Murillo et al. (5) obtained similar results to the present study, with extenders such as Triladyl and even AM. However, in the case of Murillo et al. (5) cooling was only for 6 h and in the case of Vaughan et al. (3), the initial TM at 0 h was not mentioned and could have been superior to the one we observed in our study, thus affecting the total decrease in motility observed in their samples. In our study TM decreased a 13% after 48 h of conservation compared to the initial sample (54% TM at 0 h). The main difference between these three studies with the current one is the method of obtaining the sperm, as the other studies used either an artificial vagina or vaginal aspiration. In these methods, an ejaculate is obtained, complete with the secretions of the accessory glands that generate seminal plasma. Seminal plasma contributes the very distinctive rheological characteristics of the ejaculates of these species: thread formation and high structural viscosity, which make dilution of the samples very difficult (8, 9). In our study, because we obtained sperm from the deferent ducts, the sperm had not yet come into contact with seminal plasma, and so the samples do not show the characteristic thread formation of SAC ejaculates and therefore can be homogenized better with the extenders. In addition, sperm were probably able to interact to a greater degree with the components of the extenders, for example the egg yolk, facilitating the adherence or inclusion of molecules that protect the membranes at low temperatures during cooling. Moreover, Chahuayo and Paytán (14) carried out a similar study to ours, comparing T-EY and AM extenders to dilute epididymal spermatozoa. Thus, they also worked with sperm that had not had contact with seminal plasma. However, their motility results were lower than ours when cooling for 48 h at 4°C (TM: 21% and 3% for T-EY and AM, respectively) having started out with an 80% motility at 0 h. These authors do not mention the proportion of EY they added to the Tris extender and perhaps the differences in results could be attributed to a different percentage of EY or simply to a different response between individuals to cooling and/or interaction of sperm with the extender used. However, despite the different results obtained in these two studies, both the present one and that of Chahuayo show that the addition of EY is important for preserving motility in alpaca sperm.

In addition to enabling a greater contact of sperm with the extenders, due to the absence of seminal plasma, the deviated deferent ducts technique allows one to obtain sperm with progressive motility. It is known that SAC ejaculated sperm present very low or no progressive motility, with oscillatory motility being the main pattern observed (7, 23, 24). It has also been reported that the absence or presence of seminal plasma influences the patterns of llama sperm motility (25, 26). It was noteworthy in the current study that despite starting off with a sample that had PM, the aliquots that were diluted in AM adopted an oscillatory pattern of movement, mainly after 48 and 72 h of cooling, with this pattern of motility constituting the highest proportion of the TM observed at both these evaluation times. Although the reason for this change in motility pattern is unknown, it could be related to small particles of the extender adhering to the sperm membrane and limiting their movements. This change was not observed in the samples cooled with AM-EY, despite the basic extender being the same, so possibly this difference could be attributed to the presence of EY in the medium, as it has been hypothesized that egg yolk would induce llama sperm, obtained by electroejaculation, to attain progressive motility (11, 27). Likewise, Miceli et al. (28) observed that specific surface proteins on human spermatozoa were phosphorylated to a greater extent in sperm incubated with TEST buffer with egg yolk (TYB) than those incubated in other media without EY. Results of this study also indicated that TYB proteins could also be phosphorylated by the spermatozoa during the incubation and attach to the sperm membranes. Taking into account that initiation and maintenance of progressive sperm motility seem to be regulated by protein phosphorylation (29, 30), it would be necessary to conduct studies in SAC sperm to evaluate if the mechanism of egg yolk inducing PM is through the phosphorylation of proteins.

Regarding viability, we observed between 43 and 45% live sperm in all extenders at 48 h of conservation at 5°C. These percentages signify only a 10 to 15% decrease in viability compared to time 0 h and a 20 to 24% when compared to the collected samples. This is relevant because when planning an AI protocol, the percentage of live spermatozoa is essential. Most of the studies that compare different extenders in alpaca semen focus on evaluating sperm motility, rather than viability. Chahuayo and Paytán (14) obtained lower viability results than those observed in the present study: 4 and 20% live sperm after 48 h of cooling in AM and T-EY, respectively, signifying in this case, a 95 and 77% decrease, respectively compared to their time 0 h. As far as other species of SACs, such as llamas, Giuliano et al. (2) published similar results to the current study but with a non-commercial extender composed of lactose and 20% egg yolk, observing 48% live sperm after 24 h of cooling to 5°C. Carretero et al. (31) reported slightly higher values (55–60% viability) when cooling llama sperm for 24 h with and without seminal plasma in a lactose based extender with EY. Although no reports of cooling llama sperm using commercial extenders have been published, it would be interesting to do so to be able to compare between phylogenetically close species and perhaps even make some extrapolations.

Regarding acrosome integrity, results in this study were similar to those reported for alpaca sperm obtained by artificial vagina and diluted in commercial and non-commercial extenders, where no significant differences were observed in acrosome integrity at the evaluation times studied (12). Again, this is relevant when planning an AI protocol. In addition, in this study this parameter was evaluated in conjunction with membrane function using a simple technique that is applicable in the field and which has previously been used to evaluate equine, porcine, donkey and llama sperm (21, 32, 33). The fact that the technique is simple and uses inexpensive solutions and dyes that are easy to acquire, make it possible to use it in AI campaigns in the field. Furthermore, as the samples are fixed, it allows them to be partially processed on site and evaluation can be finished once in the laboratory. This versatility and the good results obtained with this technique make this an interesting tool to implement when working with these species and under conditions that many times are far from ideal.

The percentage of normal spermatozoa in the samples diluted in any of the extenders was not altered by the cooling process. Similarly, Fumuso et al. (11, 27) observed that sperm morphology was conserved in llama frozen-thawed samples. Both these results and the ones from this study would seem to indicate that cryopreservation of SAC sperm does not affect sperm morphology in these species, at least not significantly.

Finally, sperm chromatin condensation was not affected over time with any of the extenders assayed nor did this parameter differ from that observed in collected sperm. These results contrast with those reported by Carretero et al. (31) in llama cooled sperm obtained by electroejaculation, as these authors observed a decrease in the percentage of sperm with condensed chromatin in samples cooled without seminal plasma, whereas the samples cooled with seminal plasma in the medium did not differ from raw semen. However, it is worth noting that in this latter study, the percentage of sperm with condensed chromatin in raw semen was lower than that registered in this study (74.8 vs. 91.8%) and this could have influenced the results.

## Conclusions

In conclusion, the Andromed® commercial extender with the addition of 20% EY could be an alternative option for cooling alpaca sperm obtained from deviated deferent ducts assayed in this study, not only because it preserves all sperm characteristics for 48 h of storage, but also because being a commercial extender, it is easy for the producer to prepare, requires minimum equipment and supplies and allows more repeatable results to be obtained due to its less variable composition.

Therefore, both this extender and the amount of time sperm were maintained cooled with good viability results, could provide an interesting alternative to implement in future AI protocols using alpaca sperm collected from deviated deferent ducts.

## Data Availability Statement

The original contributions presented in the study are included in the article/supplementary materials, further inquiries can be directed to the corresponding author/s.

## Ethics Statement

The animal study was reviewed and approved by Comité Institucional de Ética en Investigación (CIEI), Universidad Nacional del Altiplano Puno (UNAP).

## Author Contributions

MB carried out the study and wrote the manuscript. ET helped collect the samples. TH aided the collection of the data. DN critically read and translated the manuscript. MC designed and directed the study and critically read and corrected the manuscript. All authors contributed to the article and approved the submitted version.

## Conflict of Interest

The authors declare that the research was conducted in the absence of any commercial or financial relationships that could be construed as a potential conflict of interest. The handling editor declared a past co-authorship with the authors MC.

## Abbreviations

SAC: South American Camelids
EY: Egg yolk
AM: Andromed®
AM-EY: Andromed® extender with 20% of egg yolk
T-EY: Tris extender with 20% of egg yolk
TM: Total motility
PM: Progressive motility
CM: circular motility.
